# Endoplasmic Reticulum Stress Increases DUSP5 Expression via PERK-CHOP Pathway, Leading to Hepatocyte Death

**DOI:** 10.3390/ijms20184369

**Published:** 2019-09-05

**Authors:** Hye Jin Jo, Jin Won Yang, Ji Hye Park, Eul Sig Choi, Chae-Seok Lim, Seoul Lee, Chang Yeob Han

**Affiliations:** 1 Department of Pharmacology, School of Medicine, Wonkwang University, Iksan 54538, Jeonbuk, Korea (H.J.J.) (J.H.P.) (E.S.C.) (C.-S.L.) (S.L.); 2 College of Pharmacy, Woosuk University, Wanju 55338, Jeonbuk, Korea; 3 Brain Research Institute, Wonkwang University, Iksan 54538, Jeonbuk, Korea

**Keywords:** DUSP5, ER stress, PERK, CHOP, hepatocyte, cell death

## Abstract

Hepatocyte death is critical for the pathogenesis of liver disease progression, which is closely associated with endoplasmic reticulum (ER) stress responses. However, the molecular basis for ER stress-mediated hepatocyte injury remains largely unknown. This study investigated the effect of ER stress on dual-specificity phosphatase 5 (DUSP5) expression and its role in hepatocyte death. Analysis of Gene Expression Omnibus (GEO) database showed that hepatic DUSP5 levels increased in the patients with liver fibrosis, which was verified in mouse models of liver diseases with ER stress. DUSP5 expression was elevated in both fibrotic and acutely injured liver of mice treated with liver toxicants. Treatment of ER stress inducers enhanced DUSP5 expression in hepatocytes, which was validated in vivo condition. The induction of DUSP5 by ER stress was blocked by either treatment with a chemical inhibitor of the protein kinase RNA-like endoplasmic reticulum kinase (PERK) pathway, or knockdown of C/EBP homologous protein (CHOP), whereas it was not affected by the silencing of IRE1 or ATF6. In addition, DUSP5 overexpression decreased extracellular-signal-regulated kinase (ERK) phosphorylation, but increased cleaved caspase-3 levels. Moreover, the reduction of cell viability under ER stress condition was attenuated by DUSP5 knockdown. In conclusion, DUSP5 expression is elevated in hepatocytes by ER stress through the PERK-CHOP pathway, contributing to hepatocyte death possibly through ERK inhibition.

## 1. Introduction

Hepatocyte injury and dysfunction are crucial for the pathogenesis of liver disease progression. Hepatocyte death is a hallmark of acute and chronic liver diseases originated from various etiology including virus, drug, or metabolic stress, which contributes to the development of inflammation and fibrosis [1,2,3]. Many clinical studies have been tried to discover novel therapeutic strategies for liver diseases, regarding the intervention of parenchymal cell death in patients [2,3]. However, the patterns and mechanisms of cell death are varying on different hepatotoxic stimuli and/or stages of the diseases. Thus, the understanding of the cellular and molecular basis for hepatocyte injury in certain conditions is important for the management of liver diseases. 

Endoplasmic reticulum (ER) stress is induced by the accumulation of unfolded or misfolded proteins inside the ER, which activates the unfolded protein response (UPR) in the cells [4]. UPR is an adaptive process for maintenance of cellular homeostasis, but uncontrolled ER stress responses also account for cell damages [4]. Thus, excessive and/or sustained ER stress acts as one of the major stimuli that lead to hepatocyte injury and subsequent death [5,6]. Indeed, the pathogenesis of diverse liver diseases including nonalcoholic or alcoholic steatohepatitis, viral hepatitis, cholestasis, and drug-induced liver injury is closely associated with cellular responses against ER stress [6,7]. Nonetheless, the molecules responsible for ER stress-mediated hepatocyte injury remain largely unknown.

Dual-specificity phosphatases (DUSPs) dephosphorylate their substrates at both serine/threonine and tyrosine residues [8]. Several vital signaling molecules including mitogen-activated protein kinases (MAPKs) have been discovered as the targets of DUSPs, and their functions are involved in the regulation of biological processes such as immune responses and oncogenic transformation [8,9,10]. However, the potential roles of the specific DUSP and its regulatory basis of the gene expression, in aspects of ER stress and hepatocyte injury, are elusive.

This study investigated the effects of ER stress on the gene expression of DUSP5 and its role in hepatocyte function. Here, we report for the first time that ER stress increases DUSP5 expression in hepatocytes and the induction of DUSP5 leads to ER stress-induced hepatocyte death. To prove this, we analyzed Gene Expression Omnibus (GEO) database derived from patients with liver fibrosis and carried out animal experiments using various liver injury models. In addition, in vivo and in vitro models with ER stress were employed for the validation of the regulation of DUSP5 by ER stress. Furthermore, we performed loss- or gain-of-function experiments using hepatocytes to discover that protein kinase RNA-like endoplasmic reticulum kinase (PERK)-C/EBP homologous protein (CHOP) pathway is responsible for DUSP5 expression under ER stress condition, and the DUSP5 induction leads to hepatocyte death.

## 2. Results

### 2.1. Dual-Specificity Phosphatase 5 (DUSP5) Expression Is Elevated in Patients and Mice with Liver Diseases

**Figure 1:**
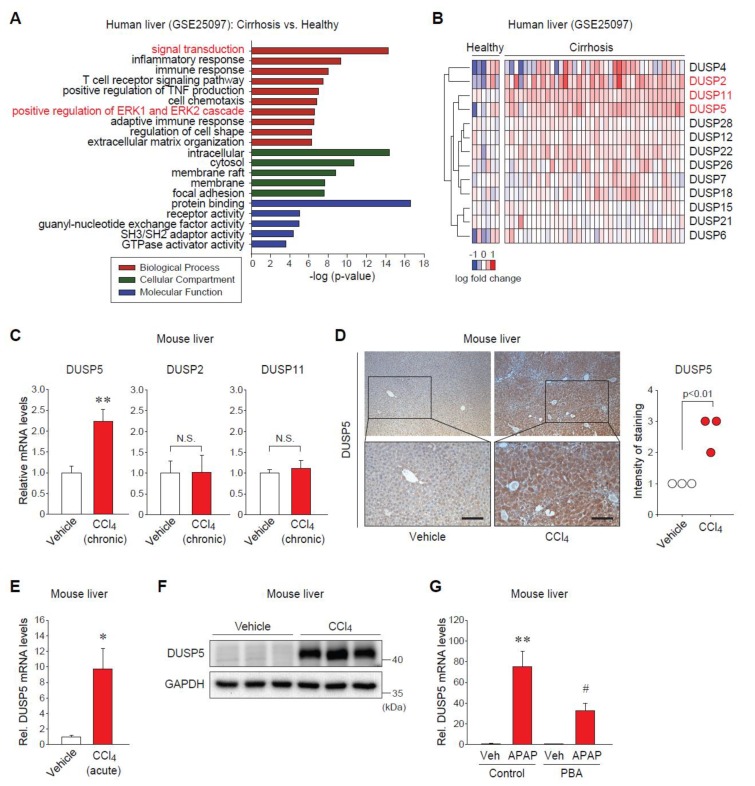
Dual-specificity phosphatase 5 (DUSP5) levels are elevated in patients and mice with liver injury (**A**) Gene ontology (GO) analysis using the DAVID bioinformatics database (derived from GSE25097). (**B**) Heatmap of the upregulated DUSPs in patients with cirrhosis versus healthy subjects (extracted from GSE25097). (**C**) Quantitative reverse transcription-polymerase chain reaction (qRT-PCR) assay for hepatic DUSPs. Mice were treated with vehicle or carbon tetrachloride (CCl_4_) (0.6 mL/kg body weight, i.p., twice a week, for 6 weeks) (*n* = 7 or 8/group). (**D**) Immunohistochemistry (IHC) for DUSP5 in the liver of mice treated as in panel (**C**). Scale bar: 50 μm. Intensity of DUSP5 staining was scored from 0 to 4 (*n* = 3/group). (**E**) qRT-PCR assay for hepatic DUSP5. Mice were given single injection with vehicle or CCl_4_ (0.6 mL/kg body weight, i.p., 24 h) (*n* = 3/group). (**F**) Immunoblotting for DUSP5 in the liver of mice treated as in panel (**E**). (**G**) qRT-PCR assay for hepatic DUSP5. Mice were treated with 4-phenylbutyrate (PBA) (100 mg/kg, i.p.) for 2 h before the injection with vehicle or acetaminophen (APAP) (500 mg/kg, i.p., 6 h) (*n* = 5/group). * *p* < 0.05 or ** *p* < 0.01 versus vehicle group (N.S., not significant); # *p* < 0.05 versus APAP-treated group.

To find a novel potential target for liver diseases, we first analyzed the public GEO database (GSE25097). Gene ontology (GO) analysis of the significantly upregulated genes in patients with cirrhosis as compared to healthy subjects showed that the groups of genes involved in intracellular signal transduction, such as the extracellular-signal-regulated kinase (ERK) pathway, were notably changed in fibrotic liver (Figure 1A). It allowed us to pay attention to the possible roles of DUSPs as MAPK phosphatases in the pathogenesis of liver injury. As a result of screening DUSPs in the dataset, three DUSPs (i.e., DUSP5, DUSP2, DUSP11) were significantly increased in the liver of cirrhotic patients (Figure 1B).

Next, we validated the alterations of those DUSPs in animal models of liver diseases associated with ER stress. Among them, only the transcript levels of DUSP5 were significantly elevated in the liver of mice treated with carbon tetrachloride (CCl_4_) for six weeks, as shown in Figure 1C, enabling us to focus on DUSP5 in the following examinations. Immunohistochemistry showed the increase in DUSP5 protein levels (Figure 1D). The effect of acute liver injury on DUSP5 expression was further examined. Consistently, both mRNA and protein levels of hepatic DUSP5 were dramatically raised in mice given a single injection of CCl_4_ (Figure 1E,F). The increases in ER stress markers were confirmed in our previous study [11]. Moreover, treatment of mice with acetaminophen (APAP) as a well-established hepatotoxic drug augmented DUSP5 mRNA levels, which was significantly attenuated by pretreatment with a chemical chaperone (Figure 1G). These results indicate that hepatic expression of DUSP5 is upregulated in liver disease states, which might be connected with ER stress.

### 2.2. Endoplasmic Reticulum (ER) Stress Increases DUSP5 Expression in Hepatocytes

**Figure 2:**
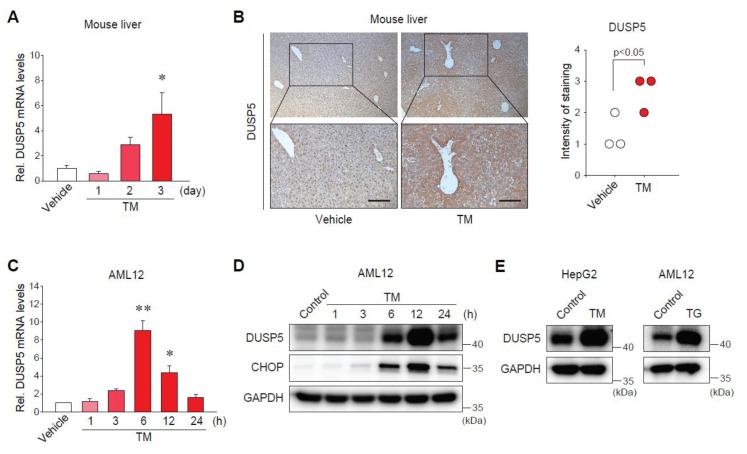
Endoplasmic reticulum (ER) stress increases the expression of DUSP5 in hepatocytes. (**A**) qRT-PCR assay for hepatic DUSP5. Mice were treated with vehicle or tunicamycin (TM, 2 mg/kg, i.p.) for the indicated time points (*n* = 4/group). * *p* < 0.05 versus vehicle group. (**B**) IHC for DUSP5 in the liver of mice treated with tunicamycin (TM) for 3 days. Scale bar: 50 μm. Intensity of DUSP5 staining was scored from 0 to 4 (*n* = 3/group). (**C**) qRT-PCR assay for DUSP5. AML12 cells were treated with TM (1 μg/ml) for the indicated time points (*n* = 4). * *p* < 0.05 or ** *p* < 0.01 versus vehicle group. (**D**) Immunoblotting for DUSP5 and C/EBP homologous protein (CHOP) in AML12 cells treated as in panel (**C**). (**E**) Immunoblotting for DUSP5 in HepG2 cells treated with 2 μg/ml of TM for 24 h (left), or AML12 cell treated with 0.3 μM of thapsigargin (TG) for 12 h (right).

To investigate whether ER stress directly regulates DUSP5 expression, we used both animal and cell models of ER stress. In the liver of mice treated with tunicamycin (TM) (a well-known ER stress inducer), DUSP5 mRNA levels were gradually upregulated (Figure 2A). The induction of DUSP5 expression was confirmed by immunostaining (Figure 2B). The ability of TM to induce ER stress was previously validated [11]. Consistently, the transcript levels of DUSP5 increased in AML12 cells in a time-dependent manner (Figure 2C), which was in parallel with the protein expression (Figure 2D). In addition, the induction of DUSP5 by TM treatment was confirmed in HepG2 cells (Figure 2E, left). Moreover, treatment of AML12 cells with thapsigargin (TG), another ER stress inducer, had the same effect (Figure 2E, right). These results demonstrate that DUSP5 expression is induced by ER stress in hepatocytes.

### 2.3. ER Stress-Induced DUSP5 Expression Is Mediated by the Protein Kinase RNA-Like Endoplasmic Reticulum Kinase (PERK)-C/EBP Homologous Protein (CHOP) Pathway

**Figure 3:**
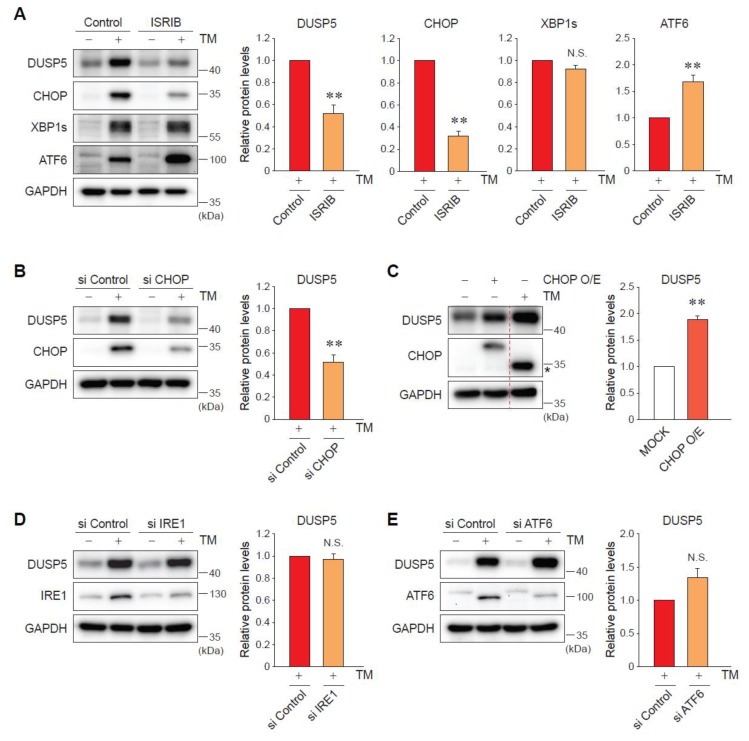
DUSP5 induction by ER stress is mediated by the protein kinase RNA-like endoplasmic reticulum kinase (PERK)-C/EBP homologous protein (CHOP) pathway. (**A**) Immunoblotting for DUSP5. AML12 cells were pretreated with 200 nM of integrated stress response inhibitor (ISRIB) for 30 min, and continuously exposed to TM for 9 h. ** *p* < 0.01 versus TM alone group (*n* = 3) (N.S., not significant). (**B**) AML12 cells were transfected with control small interfering RNA (siRNA) or CHOP siRNA for 60 h, followed by the treatment with TM for 9 h. ** *p* < 0.01 versus TM + si Control group (*n* = 4). (**C**) AML12 cells were transfected with MOCK or CHOP-overexpressing vector (Myc-flag-tagged CHOP) for 24 h, and then one MOCK group was treated with TM for 9 h. ** *p* < 0.01 versus MOCK group (*n* = 3). Asterisk presents the band of endogenous CHOP. (**D**, **E**) AML12 cells were transfected with control siRNA or each specific siRNA against inositol-requiring enzyme 1 (IRE1) (**D**) or activating transcription factor 6 (ATF6) (**E**) as in panel (**B**). (*n* = 3) (N.S., not significant).

UPR is typically mediated by three signal transducers: PERK, inositol-requiring enzyme 1 (IRE1), and activating transcription factor 6 (ATF6) [6]. To explore molecular mechanisms of DUSP5 regulation by ER stress, the effects of canonical UPR pathways were examined using a chemical inhibitor and small interfering RNA (siRNA)-mediated knockdown. Treatment with the integrated stress response inhibitor (ISRIB) (an inhibitor of PERK pathway) significantly inhibited the induction of DUSP5 by TM in AML12 cells (Figure 3A). The specific inhibition of PERK signaling, but not IRE1 (upstream for XBP1) and ATF6, was verified (Figure 3A). CHOP is a key downstream molecule of PERK and contributes to ER stress-mediated cell death [12]. Consistently, knockdown of CHOP successfully blocked the ability of TM to increase the levels of DUSP5 (Figure 3B). The overexpression of CHOP had the same effect with TM treatment, upregulating DUSP5 expression (Figure 3C). In contrast, silencing of neither IRE1 nor ATF6 affected TM-induced DUSP5 expression (Figure 3D,E). Collectively, these data indicate that CHOP downstream of PERK signaling accounts for ER-stress-mediated DUSP5 induction.

### 2.4. DUSP5 Overexpression by ER Stress Induces Hepatocyte Death

**Figure 4:**
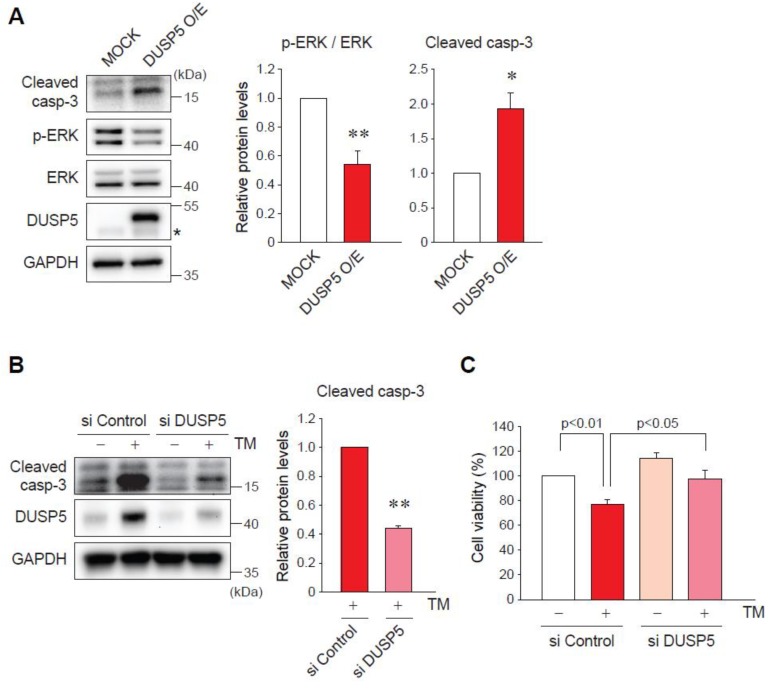
DUSP5 inhibits extracellular-signal-regulated kinase (ERK) signal and leads to ER stress-induced hepatocyte death. (**A**) Immunoblotting for cleaved caspase-3 and p-ERK. AML12 cell were transfected with MOCK or DUSP5-overexpressing vector (Myc-flag-tagged DUSP5) for 24 h. * *p* < 0.05 versus MOCK group (*n* = 3). Asterisk presents the band of endogenous DUSP5. (**B**) Immunoblotting for cleaved caspase-3 in AML12 cells transfected with control or DUSP5 siRNA, followed by the treatment with TM. ** *p* < 0.01 versus TM + si Control group (*n* = 3). (**C**) Methylthiazolyldiphenyl-tetrazolium bromide (MTT) assay in AML12 cells treated with vehicle or TM for 24 h, after transfection with control or DUSP5 siRNA. Values are the means ± standard error of mean (SEM) of 4 separate experiments (each performed in triplicate).

DUSP5 acts as a phosphatase to target ERK, thereby inhibiting its activity [9,13]. Since ERK signaling is one of the main pathways for cell survival [14], we hypothesized whether DUSP5 is associated with hepatocyte death under ER stress condition. First, we checked the possible regulation of ERK phosphorylation by DUSP5. As expected, the enforced expression of DUSP5 caused a decrease in p-ERK levels in AML12 cells (Figure 4A). Then, we determined the role of DUSP5 in ER stress-induced hepatocyte death. The activation and cleavage of caspase-3 is a key indicator of cell death exerted by ER stress [15]. The overexpression of DUSP5 raised cleaved caspase-3 expression (Figure 4A). Consistently, treatment of AML12 cells with TM increased the levels of cleaved caspase-3, which was notably inhibited by DUSP5 knockdown (Figure 4B). In addition, MTT assays showed that the decrease in cell viability by TM was significantly recovered by silencing of DUSP5 (Figure 4C). Our results suppose that DUSP5 functions as a mediator of ER stress-induced hepatocyte death possibly through ERK inhibition.

## 3. Discussion

Cellular integrity and homeostasis are maintained by the proper balance between the activation and deactivation of signals, attributing to the coordinated regulation by diverse signaling molecules and pathways. Thus, its imbalance and aberrant signal amplification is often observed in disease states and may be critical for the pathogenesis. An important finding of the present study is the identification of DUSP5 as a phosphatase changed in liver diseases and a novel potential marker of liver injury. Our analysis of human GEO database and the experiments using animal models found that the hepatic expression of DUSP5 increased in patients with liver cirrhosis, and in mice with liver fibrosis at both transcript and protein levels. Of note, DUSP5 expression was profoundly enhanced in acutely injured liver of mice exposed to liver toxicants, implying the possible detrimental roles of DUSP5 in early pathogenic events during liver disease progression. In addition, the induction of DUSP5 observed in the liver of APAP-treated mice was diminished by treatment with the chemical chaperone, which indicates that DUSP5 overexpression can be involved in drug-induced liver injury in association with ER stress. Recent reports suggested that several other DUSPs such as DUSP9, DUSP12, and DUSP26 had the protective effects on the development of nonalcoholic steatohepatitis in mice, and the expressions of those DUSPs decreased in the disease condition [16,17,18], supporting the concept that the overexpressed DUSP5 may play a role in the process of diverse liver diseases at different stages. 

Besides the pathophysiological roles of specific DUSPs, their cellular determinant and regulatory basis are largely unknown. Recently, it has been suggested that DUSP5 expression is regulated by serum response factor (SRF) and ETS like-1 protein (Elk-1) [19], and by microRNAs (i.e., miR-95 and miR-32-5p) [20,21]. Another key finding of this study is discovery of the direct regulation of DUSP5 gene expression by ER stress. Treatment of ER stress inducers upregulated the expression of DUSP5 in hepatocytes, which was validated in the in vivo model using TM. Taken together with the induction of DUSP5 expression in liver diseases, it seems that DUSP5 may contribute to the pathogenesis of ER stress-associated liver disease progression. Moreover, we newly identified the PERK-CHOP pathway, but not IRE1 and ATF6, as a signal for positive regulation of DUSP5 expression under ER stress condition. The results from the experiments using the chemical inhibitor and siRNA-mediated knockdown of UPR pathways revealed that CHOP downstream of PERK is responsible for the induction of DUSP5 by ER stress. CHOP overexpression alone even in the absence of ER stress stimulation efficaciously induced DUSP5 expression, supporting the bona fide effect of CHOP on the gene regulation of DUSP5. Our additional promoter analysis predicted that the CHOP-binding motifs [22,23] putatively exists in the proximal region of human and mouse DUSP5 gene promoters (Supplementary Figure S1), which needs to be clarified in further examinations. Our recent study showed that ER stress activated the NACHT, LRR, and PYD domains-containing protein 3 (NLRP3) inflammasome via PERK-CHOP signaling, which was inhibited by farnesoid X receptor [24]. Hence, the PERK-CHOP pathway may contribute to hepatic dysfunction through the regulation of multiple gene expression in response to ER stress.

Phosphorylation is a pivotal mode of intracellular signal transduction for the modulation of cell fate, which is tightly regulated by both kinases and phosphatases. We demonstrated in this paper that DUSP5 induction may have a role in ER stress-induced hepatocyte death probably through ERK inhibition. In this study, the inhibitory effect of DUSP5 on ERK phosphorylation was also found in a hepatocyte model, expanding our knowledge on the action of DUSP5 in the MAPK regulation. Since ERK2 is involved in the stabilization of DUSP5 [25], a signaling loop between DUSP5 and ERK may occur and probably affects cell viability. However, the complex effects of ERK on cell death has also been suggested [14,26], although it is often required for cell survival. Thus, the possible role of DUSP5 inhibition of ERK activity in ER stress-mediated hepatocyte death needs to be carefully interpreted. Furthermore, the potential other substrate(s) of DUSP5 for the regulation of hepatocyte functions should be recognized in future studies. 

In the current study, we also found that DUSP5 overexpression caused the increase in the apoptotic marker in hepatocytes, which was reciprocally changed with ERK activity. Moreover, ER stress-triggered hepatocyte death was attenuated by DUSP5 knockdown, suggesting the potential effects of DUSP5 on hepatic injury. Recent studies showed that loss of DUSP5 protected against hypertension-induced nephropathy in rates [27], and DUSP5 overexpression suppressed endotoxin-induced inflammatory gene expression in macrophages and inhibited the activity of nuclear factor-κB (NF-κB) in a phosphatase-activity independent manner [28]. Thus, the precise mechanisms and roles of DUSP5 in cell death and liver disease progression require further examinations and in vivo validation in future studies. The preventive effects of DUSP9, DUSP12, and DUSP26 on fatty liver relied on the suppression of apoptosis signal-regulating kinase 1 (ASK1) or transforming growth factor beta-activated kinase 1 (TAK1) [16,17,18]. Our previous study showed that the induction of pleckstrin homology-like domain, family A, member 3 (PHLDA3) by ER stress led to hepatocyte death via the repression of AKT [11]. Thus, the partial effect of DUSP5 knockdown on the recovery of cell viability under ER stress condition seems to be attributed to the possible involvement of PHLDA3 and other unknown molecule(s). Collectively, it is highly likely that ER stress-associated hepatocyte injury might be mediated by the overexpression of DUSP5 and following inhibition of ERK, in conjunction with the regulation of other signals including AKT. 

In conclusion, DUSP5 expression levels are elevated in chronically and acutely injured liver, and ER stress upregulates its gene expression through the PERK-CHOP pathway in hepatocytes. The induction of DUSP5 is responsible for hepatocyte injury and death under ER stress condition, which might be mediated at least in part by ERK inhibition. Our findings provide a novel insight into molecular basis for ER stress-mediated cell death and related pathogenic events of liver diseases.

## 4. Materials and Methods

### 4.1. Materials 

Anti-DUSP5 and anti-glyceraldehyde 3-phosphate dehydrogenase (GAPDH) antibodies were obtained from Abcam (Cambridge, MA, USA) and Santa Cruz Biotechnology (Santa Cruz, CA, USA), respectively. Antibodies directed against CHOP, spliced form of X-box-binding protein-1 (XBP1s), IRE1, and ATF6, phosphorylated ERK, ERK or cleaved caspase-3 were purchased from Cell Signaling Technology (Danvers, MA, USA). Horseradish peroxidase-linked anti-rabbit and anti-mouse IgGs were also provided from Cell Signaling Technology. TM, ISRIB, and other reagents were supplied from Sigma (St. Louis, MO, USA). 

### 4.2. Bioinformatic Analysis 

Hepatic gene expression data for patients with fibrosis were extracted from the Gene Expression Omnibus (GSE25097). Differentially expressed genes (DEGs) were chosen as the genes with statistical significance (*p* < 0.01 with a fold-change over 1.5) in cirrhotic patients as compared to healthy subjects, and clustered by GO analysis using the Database for Annotation, Visualization and Integrated Discovery (DAVID) 6.8 bioinformatics resource (https://david.ncifcrf.gov/), and the enriched terms were presented as top ten biological process (BP) and each top five cellular compartment (CC) or molecular function (MF) category. Upregulated DUSPs in cirrhosis patients were visualized using heatmap, and the significantly changed (*p* < 0.05 with a fold-change over 1.5) DUSPs were marked in red. 

### 4.3. Animal Treatments

The animal samples used in the present study were derived from the previous studies [11,29]. Animal experiments were performed according to the guidelines of the Institutional Animal Care and Use Committee at Seoul National University. C57BL/6 mice were purchased from Charles River Orient (Seoul, Korea). For chronic liver damages, six-week-old male mice were intraperitoneally (i.p.) injected with vehicle or CCl_4_ (0.6 mL/kg) twice a week for 6 weeks. For acute liver injury, eight-week-old male mice were given a single injection with vehicle or CCl_4_ (0.6 mL/kg, i.p.) for 24 h. In another experiment, eight-week-old male mice were administered intraperitoneal injection of vehicle or APAP (500 mg/kg, 6 h) after pretreatment with vehicle or 4-phenylbutyrate (PBA, 100 mg/kg, 2 h). For ER stress model, six-week-old male were given a single injection with vehicle or TM (2 mg/kg, i.p.) for 24–72 h.

### 4.4. RNA Isolation and Real-Time Reverse Transcription-Polymerase Chain Reaction (RT-PCR) Assays

Total RNA was extracted using TRIzol (Thermo Fisher Scientific, Waltham, MA, USA), and 1 μg of total RNA was reverse-transcribed to obtain cDNA. For quantitative analysis, the cDNA samples were diluted 1:3 with RNase-free water and 2 μL of this dilution was used. Quantitative RT-PCR (qRT-PCR) was performed using SYBR Premix Ex Taq II kit (Takara Bio, Shiga, Japan) and StepOne instrument (Applied Biosystems, Thermo Fisher Scientific) according to the manufacturer’s instructions. A melting curve of each PCR amplicon was determined to validate its accuracy. The relative levels of each mRNAs were normalized to β-Actin and calculated using the 2^−∆∆Ct^ method. Primer sequences and annealing temperatures for PCR are provided in Supplementary Table S1. 

### 4.5. Immunohistochemistry 

Immunostaining was done similarly as described in the previous study [11]. Briefly, the paraffin-embedded tissue sections were deparaffinized with xylene and rehydrated with alcohols series. The sections were interacted with anti-DUSP5 antibody (Thermo Fisher Scientific) overnight at 4 °C, followed by incubation with polymeric horseradish peroxidase (HRP)-linked secondary antibody. The labeling was done using 3,3′-diaminobenzidine. The images were obtained using light microscope (Olympus, Tokyo, Japan), and the areas were randomly selected in a consistent and unbiased manner, and whole areas of each slide were examined to verify the quality of staining. Among the multiple pictures, the representative image was shown. The staining intensities were measured using ImageJ software.

### 4.6. Cell Culture 

AML12 (non-transformed mouse hepatocyte-derived cell line) and HepG2 (human hepatocyte-derived cell line) were purchased from American Type Culture Collection (ATCC) (Rockville, MD, USA). AML12 cells were cultured in the Dulbecco’s Modified Eagle Medium (DMEM)/F-12 containing 10% fetal bovine serum (FBS), insulin-transferrin-selenium X (ITSX), dexamethasone (40 ng/mL), 100 units/mL penicillin, and 100 μg/mL streptomycin. HepG2 cells were maintained in the DMEM containing 10% FBS, and the antibiotics. The cells with less than 20 passage numbers were used. 

### 4.7. Immunoblottings

Immunoblot analysis was carried out according to the previously published methods [11]. Cells were centrifuged at 3000× *g* for 3 min, and then lysed in lysis buffer in the ice for 30 min. The lysates were centrifuged at 10,000× *g* for 10 min to attain supernatants. Proteins of interest in lysates were resolved using polyacrylamide gels and transferred to nitrocellulose or poly(vinylidene fluoride) membrane. The bands were developed using enhanced chemiluminescence (ECL) system (Millipore, Billerica, MA, USA). The band intensities were quantified using ImageJ software and normalized to GAPDH. 

### 4.8. Transient Transfection and Small Interfering RNA (siRNA) Knockdown

AML12 cells were transfected with MOCK or overexpression constructs (0.5 or 1 μg), or with control siRNA or each specific siRNAs (100 pmol) using Lipofectamine 2000 (Invitrogen, Thermo Fisher Scientific) according to the manufacturer’s instructions. Myc-flag-tagged CHOP (NM_007837) and Myc-flag-tagged DUSP5 (NM_001085390) open reading frame (ORF) clones were obtained from OriGene Technologies (Rockville, MD, USA). Scrambled siRNA (control) and the specific siRNAs direct against CHOP, IRE1, ATF6, or DUSP5 were purchased from Thermo Fisher Scientific. 

### 4.9. Methylthiazolyldiphenyl-tetrazolium bromide (MTT) Assay 

TM-induced cytotoxicity was assessed, as previously described [11]. Briefly, AML12 cells were plated at a density of 2 × 10^5^ cells per well in a 24-well plate to measure the degree of cell survival. After treatment, viable cells were stained with MTT reagent (0.25 mg/mL, 1 h). Then, the media were removed, and formazan crystals produced in the wells were dissolved in dimethylsulfoxide. Absorbance was measured at 570 nm using a microplate reader (Molecular Devices, San Jose, CA, USA). 

### 4.10. Statistical Analysis 

Data were presented as mean ± standard error of mean (SEM) and statistically significance was calculated by the Student’s t-test or by the one-way analysis of variance test with a post-hoc multiple comparison procedure. Significant differences were considered at *p* < 0.05 or *p* < 0.01. Statistical analyses were performed using IBM SPSS Statistics 24 software. 

## Supplementary Materials

Supplementary materials can be found at https://www.mdpi.com/1422-0067/20/18/4369/s1. Figure S1. Putative CHOP-response elements (CHOP-REs) in human and mouse *DUSP5* promoters. Table S1. The primer sequences for qRT-PCR assays.

## Abbreviations

APAP	Acetaminophen
ATF6	Activating transcription factor 6
CCl_4_	Carbon tetrachloride
CHOP	CCAAT-enhancer-binding protein homologous protein
DUSP	Dual-specificity phosphatase
ERK	Extracellular-signal-regulated kinase
ER stress	Endoplasmic reticulum stress
GAPDH	Glyceraldehyde 3-phosphate dehydrogenase
GEO	Gene Expression Omnibus
GO	Gene ontology
IRE1	Inositol-requiring enzyme 1
MAPK	Mitogen-activated protein kinase
PBA	4-phenylbutyrate
PERK	Protein kinase RNA-like endoplasmic reticulum kinase
TG	Thapsigargin
TM	Tunicamycin
UPR	Unfolded protein response
XBP1	X-box-binding protein-1
