# Supplementary material for: Endoplasmic Reticulum Stress Increases DUSP5 Expression via PERK-CHOP Pathway, Leading to Hepatocyte Death
Source: Int J Mol Sci. 2019 Sep 5;20(18):4369. doi: 10.3390/ijms20184369 (PMC6770509; doi:10.3390/ijms20184369)
Supplement: Supplementary file 1 [file ijms-20-04369-s001.zip › Suppl Table 1_DUSP5_rev-2nd.pdf]

Supplementary Table 1. The primer sequences for qRT-PCR assays

Species	Gene names	Pairs	Primer sequences (5'-3')	Annealing temperatures for PCR (℃)
Mouse	DUSP5	Forward	TCGTGCTGGACCACGGTAG	58
		Reverse	CTGAGAACGGGCTTTCCACA	
	DUSP2	Forward	TATGACCAGGGTGGTCCTGT	58
		Reverse	GGCACTGATCTCCACCATCT	
	DUSP11	Forward	CACCAACAACAAACCTGTGAA	58
		Reverse	CCTCGCACTCCAGAAGAATC	
	β-Actin	Forward	CTGAGAGGGAAATCGTGCGT	58
		Reverse	TGTTGGCATAGAGGTCTTTACGG	
