# Supplementary figures and images for: Endoplasmic Reticulum Stress Increases DUSP5 Expression via PERK-CHOP Pathway, Leading to Hepatocyte Death
Source: Int J Mol Sci. 2019 Sep 5;20(18):4369. doi: 10.3390/ijms20184369 (PMC6770509; doi:10.3390/ijms20184369)

Putative CHOP-REs

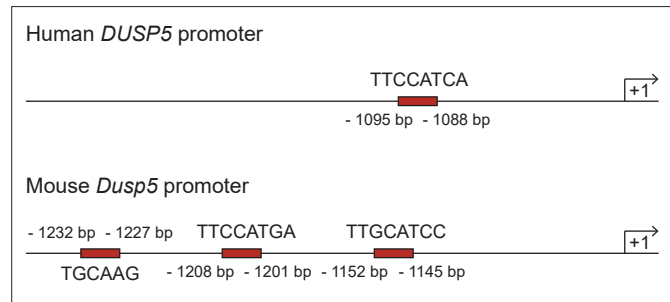

**Supplementary Figure 1**

Supplement: Supplementary file 1 [file ijms-20-04369-s001.zip › Suppl Figure 1_DUSP5.pdf]
